# Conceptual model for nurse educators to facilitate their presence in large class groups of nursing students through reflective practices: a theory synthesis

**DOI:** 10.1186/s12912-022-01095-7

**Published:** 2022-11-16

**Authors:** Kathleen Froneman, Emmerentia du Plessis, Anneke Catherina van Graan

**Affiliations:** Faculty of Health Sciences, School of Nursing Science, North-West University, Private Bag X1290, Potchefstroom, 2520 South Africa

**Keywords:** Large class settings, Model, Nurse educators, Nursing students, Presence, Reflective practices

## Abstract

**Background:**

Nurse educators are required to be present and reflective while directly involving nursing students in teaching–learning using creative and innovative interaction. Heavy daily workloads (including the teaching of large classes, clinical supervision and research) could hamper facilitating their presence through reflective practices. There is insufficient information on how nurse educators can facilitate their presence through reflective practices in large class groups of nursing students.

**Methods:**

The researchers followed Walker and Avant’s strategy of statement and theory synthesis to develop this model. Three iterative steps to theory synthesis involved identifying, defining, and classifying main and related concepts, defining relational statements, and organising the main and related concepts, relational statements, and the conceptual framework into an integrated and efficient representation. This was done by reviewing the literature. Conclusion statements were formulated using statement synthesis.

**Results:**

A model to facilitate the presence of nurse educators in large class settings using reflective practices was developed as a theoretical framework to guide teaching–learning practices. Six conclusion statements emerged on the theoretical constructs presence and reflective practices of nurse educators.

**Conclusions:**

The model addresses the gap in the literature and contributes substantially to deepening the body of knowledge in the nursing education domain of South Africa and internationally, to serve as a model for guiding nurse educators in their teaching–learning practices.

## Background

Various models are available in the nursing science literature focusing purely on improving the presence of the nurse [1, 2] and the nurse as a reflective practitioner [3]. McMahon and Christopher [2] developed a mid-range “Theory of Presence” whereby strategies were identified to facilitate presence skills in the undergraduate curriculum. In addition, the literature reveals conceptual frameworks from various authors on reflection. In reforming education, Dewey [4] founded the model of reflective inquiry that emphasised what it means to think reflectively and achieve personal learning. The theory on reflective practices built on the work of Dewey by Schőn [5] that links reflection to professional development and professional practice. The work of Boud et al. identified that for reflection to be a valid way of learning, emotions are required [6]. Furthermore, the American sociologist Mezirow [7] developed transformative learning theory to facilitate the learning and transformation of adults in the business environment; this describes how people develop and use critical self-reflection to consider their beliefs and experiences. Black and Plowright [8] developed a multi-dimensional model of reflective learning that has contributed to the understanding of reflection for learning with a focus on professional development.

No model has yet been developed for nurse educators that focuses on “presence” and “reflective practices” in the teaching–learning context of large classes. Yet nurse educators are required to implement creative, innovative, and reflective ways of interacting with and involving nursing students in the teaching–learning process and to facilitate presence [9–11]. In particular, nurse educators at accredited Nursing Education Institutions (NEIs) in the North West Province who teach large classes need guidance on building presence and reflective practices as teaching–learning strategies; such a model will provide a theoretically grounded schematic framework to apply in teaching–learning situations [12].

COVID-19, has resulted in a blended teaching–learning approach including large online (virtual) classes. Such remote learning could hinder the facilitation of presence through reflective practices. Nurse educators also face heavy workload demands, including teaching of large classes, clinical supervision, and research activities [11, 13]. These educators may also have insufficient knowledge and confidence regarding alternative teaching approaches and may experience time demands for designing, testing and implementing and new teaching approaches; selecting and grading assessments; they may also suffer from feelings of discomfort and anxiety [10, 11]. Teaching large class groups requires nurse educators to implement more interactive teaching–learning strategies that optimise opportunities for student engagement in the content as well as the learning process [9]; such optimisation will enhance the standard and quality of nursing education [14–16]. Thus, the research question arises: *What does a practice model to guide nurse educators at accredited NEIs with large class settings to facilitate presence in nursing students through reflective practices as a teaching–learning strategy entail?*

## Research aim

The aim was to develop a model for nurse educators at accredited NEIs with large classes; the model is intended to facilitate the presence of nurse educators as they teach nursing students using reflective practices as teaching–learning strategies.

## Methods

A theory-generative design was followed for model development. The model was conceptualised, structured, and contextualised through statement and theory synthesis as proposed by Walker and Avant [17]. The purpose of theory synthesis is to develop the model which is an interrelated system of ideas from evidence where concepts and statements are organised into a network or whole [17]. The process of theory synthesis consists of three iterative steps. During Step 1, the main and related concepts were identified, defined, and classified which served as anchors for the synthesised model. In Step 2, the literature was reviewed to identify and define the main and related concepts and to specify the nature of the relationships between them. Lastly, in Step 3 the main and related concepts, relational statements and conceptual framework were organised into an integrated and efficient representation (that is, the model). Finally, conclusion statements were developed for the two theoretical constructs “presence” and “reflective practices”. Repeated words, ideas and phrases were categorised together through statement synthesis to develop the conclusion statements to strengthen the significance of the model.

## Results

**Fig. 1:**
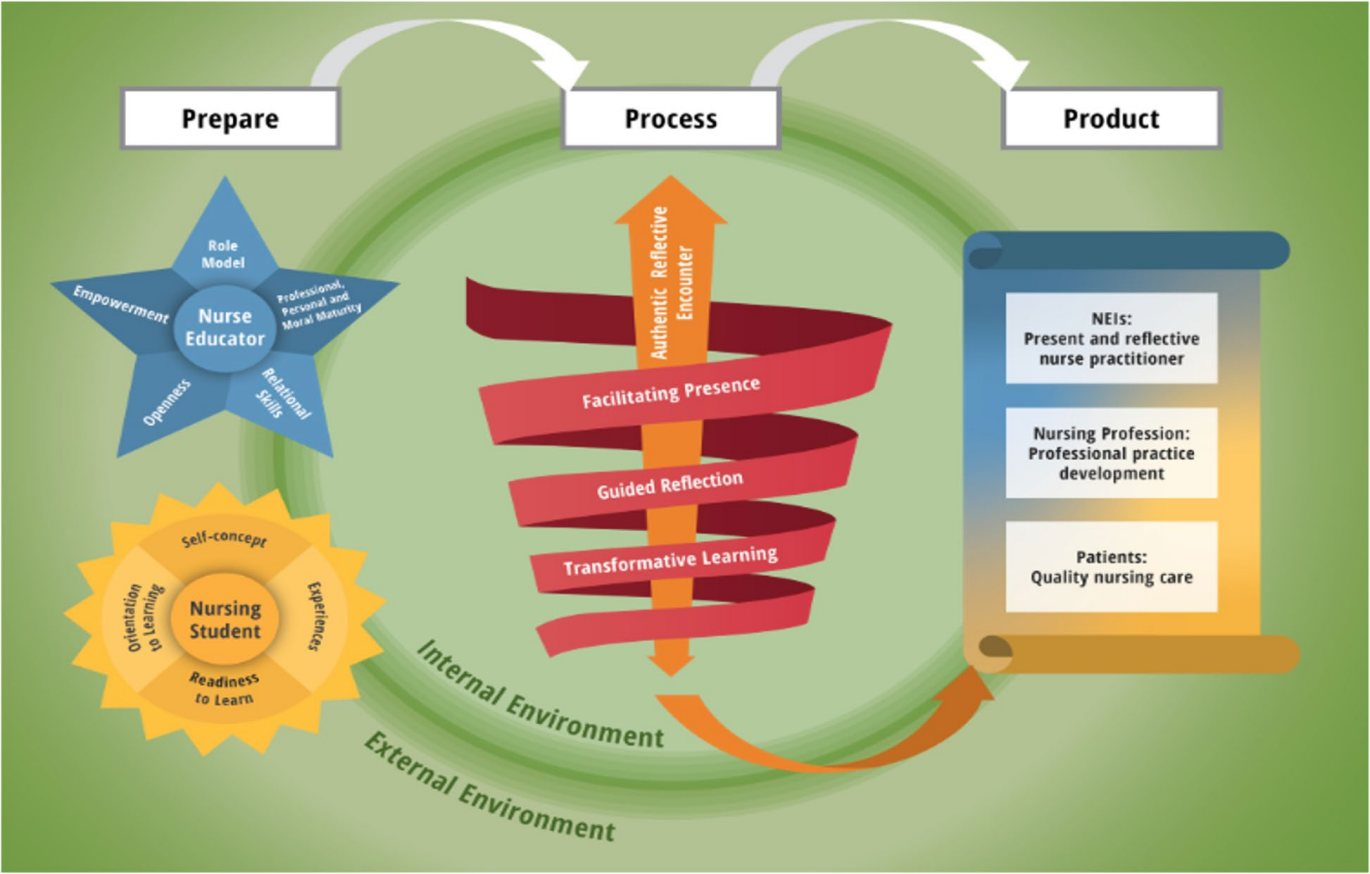
A model for nurse educators to facilitate presence in large class group settings through reflective practices

The model (Fig. 1) was structured and contextualised based on the six questions of Chinn and Kramer to ensure a complete description of the model was formed [18]. The description of the model included 1) statement of the purpose; 2) identifying the assumptions of the model; 3) clarifying the context; 4) clarifying of the structure (identified, defined and classified main and related concepts, followed by design of relational statements); and 5) the process description of the model [18].

### The purpose of the model

The purpose of this model was to provide nurse educators with a theoretically grounded schematic framework to be implemented at accredited NEIs in the setting of large classes to facilitate their presence for nursing students through reflective practices used as teaching–learning strategies.

### Identifying assumptions of the model

Assumptions are the basic underlying premises from and within which theoretical reasoning proceeds. These influence all aspects of structuring and contextualising a model [18]. The assumptions address the basic truths that are believed to underlie theoretical reasoning and will direct the reader as to what the researchers accepts as the truth. The assumptions of the model are informed by pragmatism, the “theory of presence” [19] and the model for “promoting reflection in learning” [6]. The model for nurse educators to facilitate their presence in nursing students through reflective practices at accredited NEIs in the context of large class group settings is based on the following six assumptions, derived for this study by the researchers:

- Nurse educators and nursing students are multi-dimensional (physiological, psychological and spiritual) human beings with unique experiences who interact with others and their environment to create reality and together discover (and reflect on) presence in the moment.
- Nurse educators must be receptive and dedicated to nursing students and enter the relationship with openness, patience, respect and loyalty.
- Nurse educators and nursing students are constantly interacting in an ever-changing world based on experiences. Nursing students’ reactions to learning experiences are influenced by past experiences and the way they perceive the world. They react differently to each learning experience because of different backgrounds and diversity.
- Nursing students encounter practical problems in practice and first apply critical thought by considering past experiences before acting and linking them to knowledge.
- Nurse educators assist nursing students to manage practical issues by reflecting in different ways at different times and in different contexts maintaining the view that learning objectives are not seen as something that is fixed in advance and which is strictly defined but relates to the development of the other person.
- The model is used in this study in the teaching–learning context of large classes of nursing students and is therefore context-bound.

### The context of the model

Contextual placements describe the circumstances within which the model is expected to be empirically relevant [18]. Clear statements about the context are needed to ensure that the model will be useful for practice [18]. In the context of this model, an NEI provides education and training for nursing students as regulated by the Nursing Act 33 of 2005 [20]. According to R173 of 2013 of the Nursing Act, 33 of 2005, an NEI is an institution with the capacity to offer a prescribed nursing programme upon compliance with the prescribed accreditation requirements, criteria and standards of the South African Nursing Council (SANC) for nursing education and training. They can include a university, university of technology, nursing college or nursing school, as well as health establishments [21]. The teaching–learning context consists of large classes of nursing students enrolled in four-year undergraduate nursing programmes at accredited NEIs. Structured teaching occurs in classroom and clinical practice with conditions and settings conducive to knowledge, skills and values necessary for nursing. The transfer of information and skills occurs through exposure to practical experiences. The “teaching and learning environment” for nursing students are thus equipped with needed competencies through interaction with different learning opportunities that enhance the integration of theory and practice. A blended teaching–learning approach by nurse educators facilitates their presence through reflective practices which they integrate into their nursing practice despite the large class sizes; this ultimately improves the nursing care that nursing students and graduates provide.

### The structure of the model

The structure of the model is based on the conceptual definitions of the main and related concepts and the relational statements [18] as identified during the concept analysis (Step 1 of the study method).

#### Conceptual definitions of the main and related concepts

The conceptual definition formulated for the main concept “facilitating presence through guided reflection for transformative learning” was developed through considerations outlined below.

“*Facilitating presence is leading and assisting the progress of making practicing presence possible, easier and more likely to happen. This is done through (a) making one’s character, appearance and manner felt by being in a place and being with the other holistically in a caring and compassionate manner; and through (b) an interpersonal, reciprocal, co-constructed and intersubjective process that is intentional, deliberate and considered in which the parties relate to one another through socialising, interacting, communicating and actively listening and intimately connecting in the moment where it is safe to share needs, leading to the ability to learn to see what is important to the other, respond with compassion, respond with the next best step by doing what can be done and/or being who the other needs one to be. Facilitating presence requires expertise, craftsmanship, openness, adaptation, vulnerability, intimacy, holism, sensitivity, subtlety, practical wisdom, loving fidelity, alertness, awareness, receptivity and a supportive environment and has therapeutic value. This is achieved through guided reflection where the nurse educator leads, influences, directs, accompanies, and supervises nursing students to engage in serious and careful thought using critical thinking skills by reflecting on their state of being through critical examination of experiences that results in self-inquiry, self-reflection, self-analysis and self-evaluation and in changed perspectives, a better understanding, learning and improved practice. Transformative learning, changed perspectives, gives a better understanding and creating a shift in students frame of reference, with a positive change in thoughts, feelings, beliefs and behaviour as a nurse in a present way. Deep, constructive, meaningful learning generates a complete change in nursing students’ state of*”.

#### Designing relational statements

Relational statements were deductively formulated to structurally interrelate the main and related concepts of the model which contained several levels of relational statements comprising a reasonably complete explanation of how these concepts interact [18]. The process of designing the relational statements with specific attention to the substance, direction, strength, and quality of interactions which occurred between the main and related concepts was followed [18]. The relational statements developed are as follows:

- The goal of the nurse educator is to educate nursing students to become present and reflective nurse practitioners in the nursing education context of large class settings comprising external and internal environmental elements.
- The nurse educator facilitates presence by establishing a mediated teaching–learning environment by creating an atmosphere that supports purposeful inquiry and meaningful collaboration and encourages interactive participation where nursing students can feel safe, trusted, supported, respected and free to participate.
- The nurse educator establishes meaningful relationships based on mutual trust, honesty, and dignity by connecting and sharing learning experiences with nursing students.
- The nurse educator poses specific attributes of being a role model; demonstrating professional, personal, and moral maturity; practising relational skills; being open; and empowering nursing students in facilitating presence.
- The nurse educator facilitates presence by regarding nursing students as adult learners who are self-directed with a life-long orientation to learning and who are motivated to learn, and who experience needs and interests that learning will satisfy.
- Facilitating presence through guided reflection for transformative learning is driven by the mutual need for an authentic encounter between the nurse educator and nursing students for developing an interpersonal connection.
- The nurse educator facilitates presence in nursing students by being physically, psychologically, and mentally present with nursing students. The nurse educator remains present with the large class groups of nursing students by being attentive and aware of their needs and demonstrating consideration towards their feelings.
- The nurse educator guides nursing students towards reflection through leading, influencing, directing, accompanying, and supervising learning during the acquisition of knowledge through study in the classroom, or skills acquired through experience in practice.
- During the authentic reflective encounter, the nurse educator and nursing students connect and interact in a reciprocal process.
- Transformative learning is achieved when nursing students demonstrate a positive change in their thoughts, feelings, beliefs, and behaviour resulting in transforming the way they learn in the classroom and act in practice.
- The transformative learning contributes to a positive change in the NEI by enhancing professional and personal development and satisfaction as well as improved physical and mental well-being for the nurse educator and nursing students and improved learning outcomes and positive learning experiences for nursing students that will lead to producing present and reflective nurse practitioners.
- The nursing profession will benefit through professional practice development by increasing the physical and mental well-being of the nurse and patient, improving interpersonal communication as well as meaningful relationships between the nurse and patient, and increasing professionalism and enhanced clinical knowledge for the nurse.
- The patients will benefit through increased patient satisfaction, improved patient outcomes and, ultimately, improved quality of nursing care.

Each symbol, colour and connecting line in this model was carefully chosen to convey the correct meaning, influence and impact. The structure of the model is explained according to the structural components identified based on the survey list of Dickoff [22] and includes the activity, framework, agents, procedure, dynamics, recipients, and terminus as illustrated and applied in Table 1.

**Table 1:** Application of the structural components presented in the model

Structural components:			
***Framework: External and internal environment***			
**Meaning of the symbol**	**Meaning of the colour**	**Application in the model**	
The framework is presented by a rectangle (a geometric shape that symbolises structure and order) [23]. It is associated with rationality, practicality and conformity [23]. The rectangle is used to portray safety and containment to appear efficient, grounded and accessible to a wider audience [24]	“Green” is a cool secondary colour [25] that represents balance, renewal and growth. It is the symbol of prosperity and progress [24]. It is a healing colour associated with security [24]	The green rectangle represents the framework and includes both the external and internal environment by bringing a sense of visual balance within this environment, illustrating the dynamic influence of the environmental elements. The centre gradient moving outwards in circles illustrates the dynamic and complex nature as well as the interconnectedness between the internal and external environment. The outside represents the external environment that includes the governing acts, and mandatory rules and regulations as set out by the regulatory bodies. This provides for the legal, ethical and professional frameworks in which the nurse educator practices and must be adhered to when planning, presenting and evaluating the educational encounter. The larger inner circle represents the internal environment consisting of the mediated teaching–learning environment, meaningful relationships, attributes of the nurse educator and nursing students, the teaching–learning process as well as the outcome of the model	
***Agent 1: The nurse educator***			
**Meaning of the symbol**	**Meaning of the colour**	**Application in the model**	
The nurse educator resembles a star. “Stars” are regarded as protective and guiding symbols and are widely used as a symbol of something good and positive, and are associated with conveying positive messages [26]. In this model, the symbol of a star is associated with conveying positive messages that symbolise new beginnings, a “symbol of hope and truth” [26]	“Blue” is the colour of the ocean and the sky, resembling endless opportunities. As the saying goes, “the sky is the limit”. Blue is a cool primary colour [25] that symbolises serenity, stability, inspiration and reliability. It is associated with intelligence, responsibility, professionalism and trust [27]. The colour blue also resembles certain personality strengths	The agent is the person(s) who performs the activity. In this model, agents are nurse educators (agent 1) and nursing students (agent 2) who are co-constructers in the educational encounter. The nurse educator (agent 1) is resembled by the blue 5-pointed star because they are the first line of contact when nursing students enter the nursing profession. The nurse educator is a role model associated with this symbol of “hope and truth” for creating new beginnings and life-long learning in the lives of nursing students. When nursing students feel lost and do not know what to do or where to go, they turn to nurse educators for guidance and protection. The 5-pointed star represents the attributes of the nurse educator when practising a “way of being”. These attributes are grouped under five categories associated with each point of the star and include (1) role model, (2) professional, personal and moral maturity, (3) relational skills, (4) openness, and (5) empowerment	
***Agent 2: The nursing student***			
**Meaning of the symbol**	**Meaning of the colour**	**Application in the model**	
The sun is a symbol of power, growth and health. The sun represents new beginnings and hope and demonstrates a sense of unity and perfection [24]. Nursing students are the next generation of professionals who are a symbol of hope for new beginnings in the nursing profession	The colour “yellow” is associated with the sun and symbolises optimism, energy, joy, happiness and friendship [27]. Yellow is a warm primary colour [25] that represents happiness, hope, positivity and spontaneity	The agent is the person(s) who performs the activity. In this model, agents are nurse educators (agent 1) and nursing students (agent 2) who are co-constructers in the educational encounter Nursing students (agent 2) need to be present during the delivery of quality patient care by remaining hopeful despite being confronted with sad moments, demonstrating positivity in difficult circumstances, and the ability to appreciate happiness in the moment. The yellow sun represents nursing students as new beginnings, full of hope, demonstrating a sense of unity, happiness, positivity and remaining in the moment. Nursing students as adult learners enter into the teaching–learning process with their self-concept, past experiences, readiness to learn and orientation to learning	
***Procedure: The teaching–learning process***			
**Meaning of the symbol**	**Meaning of the colour**	**Application in the model**	
A spiral is a geometric shape with three-dimensional curves with one or more turns around a centre point. It represents transformation and moving in an anti-clockwise direction, it symbolises change and development [23]. The procedure is represented by the spiral shape to illustrate the connection and community between the nurse educator and nursing students in the teaching–learning process	“Red” is a warm primary colour [25]. The colour red is associated with physical energy, courage and will. It symbolises stability, security, action, and physical and emotional survival [27]	The red spiral represents the procedure for facilitating presence through guided reflection for transformative learning. It is a continuous and ongoing process taking place between the nurse educator and nursing students to achieve set targets. The nurse educator facilitates presence by being attentive and aware of their needs and demonstrating consideration towards their feelings. Using reflection in the educational encounter leads to transformative learning. This is achieved when nursing students demonstrate a positive change in their thoughts, feelings, beliefs and behaviour resulting in transforming the way they learn in the classroom and act in practice	
***Dynamics: An authentic reflective encounter***			
**Meaning of the symbol**	**Meaning of the colour**	**Application in the model**	
An arrow is a sign consisting of a straight line with an outward-pointing V shape at either end (indicating direction). It symbolises reaching your goals and achieving your targets. The arrow resembles the direction in which the process moves to achieve the set targets	“Orange” is a warm secondary colour [25]. It is an energetic and creative colour that stimulates action [27]. It symbolises feelings of excitement, enthusiasm, warmth and determination [27]. Orange represents strength and endurance	The dynamics are the energy that drives the process. In this model, it is a mutual need for an authentic reflective encounter between the nurse educator and nursing students where they develop an interpersonal connection. The orange upward- and down-pointing arrow resembles the direction in which the process moves and symbolises determination and endurance of nurse educators and nursing students to achieve their goals and set targets. It represents the fact that potential nurse educators have to facilitate presence through guided reflection that leads to transformational learning energetically and creatively. The energy that drives the process in this practice model is the constant authentic reflective encounter that takes place between the nurse educator and nursing students, enabling the nursing students to grow into safe, caring and reflective nurse practitioners who are present during the delivery of patient care by transforming the way they feel, think, care and act in practice	
***Recipients and terminus: NEI (producing present and reflective nurse practitioners); nursing profession (professional practice development); and patients (quality nursing care)***			
**Meaning of the symbol**	**Meaning of the colour**	**Application in the model**	
A vertical scroll is a roll of paper that varies in length and has been written on. It is used to transmit information. The scroll resembles a partly unrolled sheet of paper having a spiral form at both ends and can be attached to a wooden stick to make it easier to handle. The intention of a scroll is to be used repeatedly	The “blue-yellow” gradient colour illustrates a linear diagonal-top left to bottom right gradient representing a gradual blending from one colour (blue) to another (yellow) from colours of two different tones. “Blue” is a cool primary colour that symbolises stability, inspiration and reliability and is associated with responsibility, professionalism and trust [25, 27]. “Yellow” is a warm primary colour that represents happiness, hope, positivity and spontaneity [25, 27]	The benefits and outcome of the model embody interconnectedness. The recipient and terminus are combined into one structure. The recipients are the persons who will benefit from the activity. In this model, the NEIs (recipient 1), nursing professionals (recipient 2) and patients (recipient 3). The terminus is the outcome of this authentic reflective encounter comprising present and reflective nurse practitioners, professional practice development and quality nursing care. The blue-yellow vertical scroll represents the progressive transition of nursing students resulting from the authentic reflective encounter between the nurse educator and nursing students. It symbolises the transformation of nursing students into present and reflective nursing practitioners produced by the NEI. By increasing nurses’ awareness of presence, one can transform the way they think, care and act in practice leading to professional practice development. Qualified nurse practitioners who enter clinical practice as reflective nurse practitioners by being physically, psychologically and emotionally present with the patient will be able to understand and support patients’ needs more effectively, resulting in quality nursing care	
**Connecting line**			
***The orange up-pointed curved arrow***			
**Meaning of the symbol**	**Meaning of the colour**		**Application in the model**
The up-pointed curved arrow consists of a curved line ending with an upside-down V shape pointing in a specific direction. It symbolises reaching your goals and achieving your targets. The curved line creates familiarity, and comfort and is interesting to follow	The colour “orange” is a warm secondary colour [25]. Orange is an energetic and creative colour that stimulates action. It symbolises feelings of excitement, enthusiasm, warmth and determination [27]. The colour orange also represents strength and endurance		The orange up-pointed curved arrow illustrates the outcome of the authentic reflective encounter where the nurse educator facilitates presence through guided reflection for transformative learning. It points out the direction in which the process moves to achieve the set targets. These targeted recipients include the NEIs, the nursing profession and patients who will benefit from this activity

### Process description of the model

The model (see Fig. 1) follows a three-phased strategy of prepare (Phase 1), process (Phase 2) and product (Phase 3). The process of the model for nurse educators to facilitate their presence in large class groups of nursing students through reflective practices as teaching‬–learning strategies at accredited NEIs is outlined in Table 2.

**Table 2:** Process description of the model

Phases:
***Phase 1: Prepare***
The preparation phase involves preparation and planning for the educational encounter and includes external and internal environmental elements. The external environment represents the SANC, NEI as a higher education institution, and the clinical practice that prescribes the legal, ethical, and professional frameworks that influence and guide nurse educators’ teaching–learning practices at accredited NEIs. The internal environment exemplifies the nurse educator and nursing students as multi-dimensional (physiological, psychological, and spiritual) human beings with unique experiences interacting with each other and their environment to create reality and together discover presence in the moment and reflect on it. Phase 1: internal environment includes the teaching–learning environment, meaningful relationships, attributes of the nurse educator and nursing students
The nurse educator creates the ***mediated teaching–learning environment*** by ensuring an atmosphere that supports purposeful inquiry and meaningful collaboration and encourages interactive participation where nursing students can feel safe, trusted, supported, respected and free to participate. This environment consists of the nurse educator creating conducive physical settings and authentic surroundings by utilising adequate resources and implementing sufficient time management as follows. 1) Conducive physical settings: Ensure adequate lighting, sufficient ventilation, appropriate layout, and classroom seating arrangements. Within the clinical practice, the nurse educator establishes a conducive environment through adequate orientation and effective accompaniment of nursing students. 2) Authentic surroundings: Connect with nursing students through active participation, interaction, engagement, and meaningful feedback and provide opportunities for self-reflection and self-assessment. 3) Adequate resources: Include relevant study material (study guides, textbooks, handouts, etc.); correct equipment (Proxima, whiteboard, flip charts, manikins, etc.); adequate support (faculty, colleagues, peers, and students); effective use of technology including technological strategies; and design appropriate reflective exercises. 4) Sufficient time management: Allocate sufficient time for specific educational tasks and activities
The nurse educator establishes ***meaningful relationships*** that are open and promote mutual trust, honesty, and dignity by connecting with and attuning to nursing students. The nurse educator utilises reflection to examine and transform nursing students by creating a shift in their frame of reference through discovering new meanings and perspectives. The nurse educator aims for a positive change in nursing students’ thoughts, feelings, beliefs, and behaviour, and encourages nursing students to engage in studying to acquire specific knowledge, skills and understanding needed for nursing practice. In this process, nurse educators inspire nursing students to engage and interpret direct and active learning experiences by utilising critical reflection
Within this model, the nurse educator and nursing students enter the educational encounter, each with their specific ***attributes.*** The nurse educator needs to be enthusiastic, communicative, compassionate, sincere, and trustworthy in providing stability for nursing students during teaching and learning. The nurse educator must portray certain attributes by being a role model, exhibiting professional, personal, and moral maturity, demonstrating relational skills, being open, and empowering nursing students as follows
1. *Role model*: The modelling of presence by the nurse educator (role model) to nursing students will help them to internalise the behaviour to implement during patient care. This is done by sharing experiences and intimately connecting in the moment when it is safe to share ideas. This enables nursing students to learn what is important to the other and to respond with compassion, doing what can be done or being who the other needs one to be. Further behaviours include portraying a professional appearance by adhering to the professional dress code of the institution and profession, and the correct use of non-verbal body language
2. *Maturity:* a) Professional maturity. The nurse educator remains knowledgeable, skilled, and experienced in both theory and practice to guide nursing students through processes of knowledge construction, reflection, and discussion in preparing them for professional practice. b) Personal maturity: The nurse educator demonstrates self-awareness and self-knowing by being open-minded. c) Moral maturity: The nurse educator bases their teaching practices on understanding the importance of values and attitudes in nursing care. This is achieved through adherence to the moral principles of commitment to help by being available to nursing students and showing respect for individual differences. Moral responsibility refers to the nurse educator’s willingness to engage with nursing students to strive for excellence in nursing practice
3. *Relational skills:* The nurse educator demonstrates expertise and craftsmanship in facilitating presence by being accessible and flexible. The nurse educator acknowledges her vulnerability in having to manage large classes as well as the vulnerability of nursing students being young adults and coping with student and personal lives. The nurse educator connects with nursing students in an intimate way by being who they need her to be in the moment and being present in a holistic way. By being sensitive to and aware of nursing students’ needs, the nurse educator shows practical wisdom in adapting to the class and reaching the outcomes according to nursing students’ learning needs. The nurse educator is fully present by being alert, attentive, aware, and receptive to nursing students’ needs while engaging in active listening and demonstrating consideration towards their feelings. Further, the nurse educator is authentic and shows fidelity by keeping to deadlines and expecting nursing students to reach the expected outcomes
4. *Openness.* The nurse educator is human, honest, and open to nursing students by showing interest in their lives and experiences. The nurse educator facilitates presence through an openness to learn, change and acknowledge the perspectives of others
5. *Empowerment* The nursing educator supports and guides nursing students, making them feel valued, involving them in decision-making, actively listening to them, and reducing anxiety through continuous encouragement and motivation
Nursing students as adult learners enter the teaching–learning process with their self-concept, past experiences, readiness to learn and orientation to learning. The nurse educator acknowledges the ***attributes of the adult learner*** by regarding nursing students as adult learners who are self-directed with a life-long orientation to learning, are motivated to learn, and experience needs and interests that learning will satisfy. The nurse educator can accommodate the adult learner as follows. *Self-directed*: Utilise the study guide, use the interactive method (discussions), and provide clearly defined goals and criteria for evaluation, continuous feedback, and development of critical reflection. *Readiness to learn*: Point out the relevance and value of the application of the study content, use relevant and applicable examples from real-life situations, guide students from the known to the unknown, and encourage an attitude of questioning. *Experiences*: Utilise the experience of students, develop thinking, problem-solving and evaluation skills, develop the ability to correlate theory and practice, develop critical-evaluating thinking and critical self-reflection, and provide feedback regarding achieving the purposes of learning, motivating them to study further. *Learning orientation*: Establish a physical and psychological climate conducive to learning. Involve adult students in decisions about their learning, and plan methods and content with them. Involve the students in diagnosing their own learning needs or the gap between what they know and what they feel they need to know. Motivate students to identify learning resources and to find strategies for using these resources to achieve the learning outcomes. Support students in carrying out their learning plans, and involve them in evaluating their own learning
***Phase 2: Process***
The process phase involves the implementation of the educational encounter. The nurse educator and nursing students engage in an authentic reflective encounter to develop an interpersonal connection where presence can be facilitated using guided reflection to reach transformative learning. This *teaching–learning process* comprises three steps
***Step 1: Planning for the educational encounter***: Presence is facilitated by demonstrating care and consideration in developing the content (plan and structure the lesson plan including an introduction, the presentation and a structured conclusion); selecting appropriate learning activities such as activities for reading (assignments for nursing students to become engaged), writing (journals, both handwritten and electronic, to stimulate reflection), doing (engage nursing students in doing activities, e.g., portfolios) and telling (telling of an experience to engage nursing students in reflection); selecting suitable teaching strategies such as reflective diaries, reflective journals, mindfulness minute, video discussions, authentic scenarios, role-play, critical incidents, simulations, case studies, narratives, rubrics and portfolios; selecting correct assessment techniques by deciding on the type of assessment, e.g., formative or summative; being accessible during assessment time; providing constructive and timely feedback after each assessment; and engaging in regular follow-up for clarifying any concerns
***Step 2: Presenting the educational encounter***: In presenting the content, presence is facilitated by being and remaining present in the classroom, being attentive and aware of nursing students’ needs, and demonstrating consideration towards their feelings. The most suitable teaching–learning strategy to facilitate presence is guided reflection. Guided reflection involves the nurse educator leading, directing, and supervising nursing students’ learning during the acquisition of knowledge through study in the classroom or accompanying them during skills acquisition through experience in practice. When presenting the new material, the nurse educator needs to follow nine strategies. 1) *Engage nursing students in self-reflection by determining prior knowledge*: Provide opportunities for them to reflect on the previous teaching by sharing their ideas, feelings, and perspectives. 2) *Connect with nursing students*: Display a “way of being” by welcoming and greeting your nursing students in a caring, polite, and humorous manner. Classroom rules are set during their first encounter to ensure classroom discipline, especially in large class group settings. Know your nursing students by name to show a genuine interest in them. Clearly state the learning outcomes and the outlay of the session, in the beginning, to ensure that nursing students know exactly what is happening and expected from them. Do not call out students by their names but allow them to participate as they feel comfortable. This makes them feel like an important part of the large class group and free to participate without judgement. Involve them in sharing personal and professional experiences and interpreting real-life clinical experiences. 3) *Develop new practice insights*: Reflect on learning experiences to change nursing students’ behaviour as well as their way of thinking by making meaningful connections between their previous learning and new ideas and experiences. 4) *Develop clinical competence*: Use practical examples for integrating knowledge and skills, enabling nursing students to engage in theory–practice integration. 5) *Develop critical thinking skills*: Encourage nursing students to explore decisions, thoughts, and feelings by critically analysing, synthesising, and evaluating learning experiences. 6) *Guide towards self-discovery*: Ask questions and provide an opportunity for nursing students to answer out loud, and if correct, a mark will be allocated. If the answer is incorrect, the nurse educator demonstrates sensitivity by not telling them it is wrong, but instead guiding and directing them towards discovering the correct answer for themselves. 7) *Challenge participation in self-inquiry and self-analysis*: Guide nursing students in critically reflecting on their own positive and negative experiences while learning about nursing care through discussing their thoughts, feelings, and knowledge. Guide them in practising self-inquiry and self-awareness by utilising the reflective process. In the first phase, nursing students create awareness of uncomfortable feelings and thoughts. The second phase includes engagement in critical analysis of the situation. A new perspective on a new situation is developed in the third phase. 8) *Provision of timely and constructive feedback*: Encourage active listening and communication. Encourage nursing students to listen to what others are saying, help each other to make the experience more explicit, and share certain ways of expressing or understanding specific actions. Provide appropriate feedback after discussions and assessments. 9) *Acknowledge nursing students’ contributions*: To build their morale and to make them feel valued, praise them regularly on their achieved outcomes
***Step 3: Evaluating the educational encounter***: When evaluating the new material, the nurse educator must consider the following. 1) *Acknowledge contributions*: At the end of each lesson, the nurse educator summarises the day’s work and completes a five-minute quiz. After nursing students have completed the quiz, they exchange theirs with the colleague next to them. This exercise assists them to practice presence by valuing ideas and feedback from others. 2) *Engagement in self- and peer-assessment*: The nurse educator completes a self-assessment rubric on her teaching practices for improvements. Encourage nursing students to complete a lecturer evaluation to expose them to practices such as self-reflection, self-analysis and self-evaluation that lead to a better understanding, learning and improved practice. Transformative learning is achieved when nursing students demonstrate a positive change in their thoughts and behaviour, resulting in transforming the way they learn in the classroom and act in practice. When this happens, it will continue into the final phase of achieving the set targets
***Phase 3: Product***
The product phase involves the recipients who benefit from the outcomes of the practice model and can be grouped into three categories as outlined below
***Category 1****: NEIs*: Produce present and reflective nurse practitioners that contribute to personal and professional development, enhanced personal and professional satisfaction through feelings of making a difference in the lives of others, improved physical and mental well-being and positive learning experiences
***Category 2***: *Nursing profession*: Increasing nurses’ awareness of presence can transform the way they think, care and act in practice. This can lead to professional practice development that contributes to the physical and mental well-being of the nurse and patient. It can also result in improved interpersonal communication and build meaningful relationships between nurses and patients. Further, it can lead to improved professionalism, enhanced clinical knowledge and strengthened critical reasoning of nurses as it allows them to engage in internal dialogue; this builds their ability to think through and reflect on nursing actions and hence to provide better nursing care
***Category 3:**** Patients:* Qualified nurse practitioners who enter the clinical practice as reflective nurse practitioners by being physically, psychologically, and emotionally present with the patient will be able to understand and support patients’ needs more effectively, leading to quality patient care. This contributes to increased patient satisfaction, positive patient outcomes, and improved patient care

### Concluding statements of the model

To strengthen the model developed, concluding statements were formulated through statement synthesis for “presence and reflective practices in nursing education” as follows:

- Facilitating presence through reflective practices requires a conducive teaching–learning environment that supports purposeful inquiry and meaningful collaboration. These two elements are essential to implement by nursing students exploring and challenging practice through reflection. Various environmental factors such as conducive work settings, authentic surroundings, adequate resources, and sufficient time enhance presence through reflective practice.
- The nurse educator establishes meaningful relationships with nursing students that are supportive, respectful, and non-judgemental through being open and connecting with nursing students. The nurse educator can challenge, enable, and support them for learning to become meaningful. In turn, such relationships provide a model for nursing students on how to establish therapeutic relationships, a skill that nursing students can apply to patients.
- Attributes of presence embrace the nurse educator being a role model, having professional, personal and moral maturity, demonstrating relational skills, being open, and empowering nursing students.
- Facilitating presence through reflective practices contributes to positive learning experiences by ensuring that deep and meaningful learning occurs; this requires sufficient time, adequate resources and support from organisations, colleagues, peers and students, and the use of applicable technology.
- Presence and reflective practices are essential components for theory–practice integration in that nurse educators share personal and professional experiences with their nursing students to promote the internalisation of knowledge and skills. Such practices enable them to implement what they have learnt, and develop professional knowledge, understanding and clinical competence; these elements in turn lead to transformative learning.
- Presence and reflective practices contribute to continuous professional development and life-long learning, personal and professional satisfaction, and physical and mental well-being for the nurse educator, nursing student and the patient. In turn, this leads to improved quality nursing care, and ultimately, positive patient outcomes.

### Validation of the model

After the model was developed and contextualised, it was evaluated and refined in two steps. In Step 1, the model was evaluated by a panel with expertise in model development, reflective practices and presence. They evaluated the model using critical reflection [18], resulting in the refinement of the model. Step 2 involved an empirical phase where the model was evaluated through online semi-structured focus group interviews with nurse educators and virtual World Café sessions with 4th-year nursing students enrolled in the undergraduate nursing programme at accredited NEIs. Recommendations for improvement from the panel of experts and participants were used during the final refinement of the model. The detailed process followed during the evaluation of the model is the subject of another paper.

## Discussion and recommendations

A theory synthesis in line with Walker and Avant [17] was conducted to develop a model to bridge the gap identified in the literature, namely that available models focus purely on improving presence in nursing science or the nurse as a reflective practitioner. Until the current paper, there has been no known model that implements both “presence” and “reflective practices” in teaching–learning strategies for nurse educators interacting with large classes of nursing students.

To meet the aim of this study, the current model has been conceptualised and validated as a framework to guide nurse educators at accredited NEIs with large classes to facilitate their presence in nursing students through reflective practices as teaching–learning strategies. This paper describes the model by providing an overview, defining the purpose, identifying assumptions, clarifying the context and detailing the model structure. This model addresses the gap in the literature and substantially deepens the body of nursing education knowledge in the international and South African contexts; it thereby serves as a model for guiding nurse educators in their teaching–learning practices.

This conceptual model can be applied in nursing education, practice and research. Integration of this synthesised model into the teaching practices of nurse educators involved in undergraduate, postgraduate and continuous professional development programmes could produce present and reflective nurse practitioners by:

- 1. implementing a short-term learning programme to assist nurse educators with understanding the importance of the model and to present guidelines for implementation by nurse educators throughout their training;
- 2. presenting in-service training programmes to increase nurse educators’ awareness of presence and to enhance their knowledge and skills for facilitating presence through guided reflection for transformative learning;
- 3. integrating the model into the teaching practices of nurse educators for each year of undergraduate nursing programmes to facilitate presence throughout their training; and
- 4. creating awareness campaigns in other fields (such as education) of the importance of presence through presentations to emphasise the importance of practising presence to promote the standard of education, training and practice.

The conceptual model developed by the researchers can also be applied by nurses in practice in clinical facilities. It is suggested that: 1) all categories of nurses attend a short learning programme to create awareness of presence; 2) in-service training programmes are held to orientate all categories of nurses in the clinical setting of the practice of presence; and 3) continuous professional development programmes on presence should be made available.

This synthesised model prompts the need for further research outputs. Other venues for research into facilitating presence through reflective practices include: 1) validation of the model presented in this paper and a set of guidelines for operationalisation; 2) refinement of a model contributing to theory–practice integration; 3) the development of a similar programme to be applied in other health science disciplines; and 4) guidelines for operationalising the model within other disciplines such as education.

A limitation in the process of developing the conceptual model was limited in that the literature search was restricted to peer-reviewed journal articles written in English; relevant research the presence and reflective practices written in other languages may therefore have been missed. Despite the limitations of the manuscript, the conceptual model has laid the foundation for future research and new findings that could provide further insights into the theory of facilitating presence in large classes through reflective practices.

## Conclusions

The presence of nurse educators at North-West University has elicited the intuitiveness of nursing scholars and researchers and has created awareness of intuitiveness in the field of nursing science. It was necessary to capture insight into this phenomenon, and the newly synthesised conceptual model of the researchers provides an effective way of approaching this goal. Theory synthesis proposed and developed by Walker and Avant [17] was used as a strategy to develop the conceptual model for nurse educators to facilitate their presence in a large class of nursing students through reflective practices. This paper contributes substantially to deepening the body of knowledge in the nursing education domain of South Africa and internationally, to serve as a model for guiding nurse educators in their teaching–learning practices. The researchers’ proposed model can be used as a foundation for further research and can be utilised across nursing education, practice, and management.

## Data Availability

The datasets used and/or analysed during the current study are available from the corresponding author upon reasonable request.

## Abbreviations

HREC: Health Research Ethics Committee
NEI(s): Nursing Education Institution(s)
RDGC: Research Data Gatekeeper Committee
SANC: South African Nursing Council
